# Hallucinations in Healthy Older Adults: An Overview of the Literature and Perspectives for Future Research

**DOI:** 10.3389/fpsyg.2017.01134

**Published:** 2017-07-07

**Authors:** Johanna C. Badcock, Hedwige Dehon, Frank Larøi

**Affiliations:** ^1^Centre for Clinical Research in Neuropsychiatry, Division of Psychiatry, Faculty of Health and Medical Sciences, The University of Western AustraliaPerth, WA, Australia; ^2^Australia and Perth Voices Clinic, Murdoch University Child and Adult Psychology Service, Murdoch UniversityMurdoch, WA, Australia; ^3^Psychology and Neuroscience of Cognition Research Unit, University of LiegeLiege, Belgium; ^4^Department of Biological and Medical Psychology, University of BergenBergen, Norway; ^5^NORMENT – Norwegian Centre of Excellence for Mental Disorders Research, University of OsloOslo, Norway

**Keywords:** hallucinations, perception, psychotic experiences, ageing, cognition, prevalence

## Abstract

**KEY POINTS**

➢ Studies suggest a substantial minority of healthy older adults have hallucinatory experiences, in line with existing evidence on hallucinations in other age groups, though it is still unclear if hallucination prevalence increases or declines with age in older cohorts.
➢ Stigma attached to both hallucinations and ageing leads to considerable under-reporting of these experiences in healthy older adults and may negatively bias how professionals, family members, and the public respond.
➢ Why and when hallucinations in healthy older adults remit, persist, or progress to other clinical disorders remains poorly understood.
➢ Current evidence points to a range of factors associated with hallucinations in older adults including decline in sensory or cognitive functioning, poor sleep, and psychosocial stressors (e.g., social isolation, loneliness, and bereavement), highlighting the need for accurate assessment and tailored interventions.

Hallucinations, though common in youth and younger adults, are not the preserve of these age groups. Accumulating evidence shows that hallucinatory experiences are also present at surprisingly high rates in healthy older adults in the general community. Furthermore, stigma and misunderstanding of hallucinations, together with ageism, may lead to under-reporting of these experiences by older adults, and misdiagnosis or mismanagement by health and mental health practitioners. Consequently, improved public and professional knowledge is needed about the nature and significance of hallucinations with advancing age. The purpose of this review is to provide a comprehensive overview, and critical analysis, of research on the prevalence, psychosocial, and neurobiological factors associated with hallucinations in people aged 60 years and over. To the best of our knowledge, this is the first review of its kind in the literature. The evidence supports a dynamic conceptualization of hallucinations, in which the emergence of hallucinations is viewed as a balance between the sensory, cognitive, or social impairments accompanying advancing age and the degree to which compensatory processes elicited by these impairments are successful. We briefly summarize the implications of the literature for aged care services and interventions, and stress that far more studies are needed in this important field of research.

## Introduction

The world's population is aging, with the number of adults aged 60 years and over expected to reach 2 billion by 2050 (United Nations, 2013). Furthermore, the average life span continues to increase. In Australia, for example, from 2002 to 2012 life expectancy increased from 78.1 to 79.9 years (for men) and from 83 to 84.3 years (for women) (Australian Bureau of Statistics, 2014). As a consequence, there is a growing focus on the physical and mental health needs of older adults to ensure that healthy ageing is possible. Hallucinatory experiences are particularly significant in this regard since they can indicate the presence of mental illness but are also known to occur in healthy individuals in the general population (Sommer et al., 2010; Johns et al., 2014; Kråkvik et al., 2015).

Hallucinations are hard to pin down, with a standard definition remaining elusive (David, 2004; Larøi et al., 2012). At the broadest level, hallucinations are characterized as the abnormal perception of something not present (see Table 1 for a glossary of terms); though the genesis of these experiences seems to be far more dynamic than previously realized. Thus, rather than being passive phenomena arising from deficits in cognitive-perceptual processes, this “positive” conceptualization of hallucinations maintains that a range of active, adaptive processes are involved as well, that compensate for such deficits. Since compensatory processes have been well-studied in the context of normal ageing (e.g., Reuter-Lorenz and Park, 2014; Peelle and Wingfield, 2016), hallucinatory experiences in healthy older adults may provide a particularly valuable window on the underlying mechanisms involved. Despite this, later-onset hallucinations have mostly been examined in the context of Lewy Body disorders and dementia (Collerton et al., 2012; El Haj et al., 2015). In contrast, the diversity and significance of hallucinations in healthy older adults have received much less attention (Tien, 1991). However, new epidemiological data indicate that nearly a quarter of first onsets of hallucinations occur after the age of 40 years (McGrath et al., 2016). Such findings suggest that hallucinatory experiences in later life (i.e., in the absence of mental disease or disorder) warrant considerably more attention. Against the backdrop of an aging population, they also emphasize the need for a greater understanding of the causes, correlates, and consequences of hallucinations in healthy older individuals in order to guide the planning and provision of aged-care services.

**Table 1:** Glossary of terms and definitions.

**Charles Bonnet Syndrome:** Typically involves the experience of formed visual hallucinations, with insight that the experience is not real, in people who are otherwise mentally “normal.” Percepts may involve familiar or unfamiliar images of people, animals, inanimate objects, and patterns.
**Hallucinations:** The perception of an object or event in the absence of an external stimulus.
**Minor hallucinatory phenomena:** Include sensed presence, passage hallucinations, and visual illusions.
**Musical Hallucinations:** The subjective experience of hearing music, or aspects of music, (with or without voice and lyrics) when none is being played.
**Out-of-body experience:** Typically involves the sense that you are floating outside your body. In some cases it may involve the feeling that you are perceiving your body as if from a position outside of your own body
**Passage Hallucinations:** The experience of a stimulus moving past the perceiver, in the peripheral field.
**Sensed presence:** The vivid sensation of the presence of another known or unknown person, typically close to but slightly behind the perceiver. Sometimes referred to as the “Guardian Angel” item, or the “experience of continued presence” in reference to sensing the presence of a deceased relative by a bereaved person.
**Tinnitus:** The conscious perception of a sound when no external sound source is present; sounds are typically simple, such as ringing, hissing or buzzing but occasionally more complex percepts are reported, including music.
**Visual illusions:** Brief misperceptions of living things or objects that differ from objective reality.

The purpose of this article is to provide the first synthesis and critique of the literature on hallucinations in healthy (non-clinical) older adults. We summarize research on the prevalence, psychosocial, and neurobiological factors associated with hallucinations in samples predominantly aged over 60 years. Where relevant, we highlight the similarities and differences in our findings with the literature on hallucinations in younger and/or clinical populations, and discuss implications for aged care services and intervention. Finally, we summarize the strengths and gaps in the evidence base and outline priorities for future research.

## Methods

### Methodological difficulties and search process

Initial scoping of the literature revealed a number of difficulties in attempting to conduct a systematic review of all the evidence on hallucinations in healthy older adults. Hallucinations can occur at any age and studies do not always report results by age group. Limiting the search to studies of healthy samples only, missed many reports which focused on clinical groups/disorders but were then further divided into clinical and non-clinical categories. Restricting the search to current or recent evidence resulted in an under-representation of research in less popular, but significant, areas (such as bereavement), whilst limiting the search term to “hallucinations” missed studies with a focus on specific experiences such as tinnitus, Charles Bonnet Syndrome, or sensed presence. In view of the large variety of aims, methods, and perspectives in research on hallucinations in older adults we synthesized the literature into three major themes, presented below (prevalence and characteristics; stigma and distress; factors associated with hallucinations in older adults). In order to focus on hallucinations in healthy individuals, studies on abnormal perceptual experience in older adults with dementia, neurological disease (e.g., tumor, epilepsy, Parkinson's), delirium, or psychotic disorder were excluded. The review covers a wide spectrum of experiences, including complex, simple, and minor hallucinations (such as sensed presence and passage hallucinations) in any modality.

## The prevalence and characteristics of hallucinations in older adults

Very few studies have directly and specifically examined the prevalence (and other aspects) of hallucinations in the elderly, non-clinical population. Either studies simply do not include older age groups (e.g., older than 60 years), or they do, but combine all age groups into one or do not report specific prevalence rates for each age group. Nevertheless, in general, there seems to be a significant minority of this population that has these experiences—which is in line with recent conclusions regarding the experience of hallucinations in younger, non-clinical populations (Jardri et al., 2014; Johns et al., 2014). However, the documented prevalence rates of hallucinations in older adults vary between 0.4 and 37%. For example, Turvey et al. (2001) report 20%, Cole et al. (2002) report 2.5%, Lyketsos et al. (2000) report 0.6%, Larøi et al. (2005) report 37%, Geda et al. (2008) 0.4%, Okura et al. (2011) 2.2%, Kråkvik et al. (2015) 3.7%, and Subramaniam et al. (2016) 2.7%. A summary of important details regarding these studies (e.g., age of participants, particular characteristics of participants, how hallucinations were assessed, whether only hallucinations alone, or combined with other experiences, etc.) is provided in Supplementary Table 1, which have undoubtedly contributed to the variability of these estimates (see Pang, 2016 for variations in reported prevalence of Charles Bonnet Syndrome). Of these differences between studies, how hallucinations were assessed is most probably the most important characteristic that explains this large variability in prevalence rates and, in particular, which timeframe was adopted. For instance, the highest rate (37%) is reported in Larøi et al. (2005) where items from the LSHS were used—and where the timeframe is lifetime prevalence (e.g., “In the past, have you ever had the following experience?”). In contrast, the lowest prevalence rates are reported in studies (e.g., Lyketsos et al., 2000; Cole et al., 2002; Geda et al., 2008; Okura et al., 2011) where a 1-month timeframe (many of these studies use the NPI to assess hallucinations) is utilised (“Have you had the following experience in the past month?”) and thus large differences in prevalence rates are to be expected.

For those studies that included prevalence rates for specific age groups, the results seem to suggest that prevalence rates of hallucinations decrease with age, although this is not always the case. For example, drawing on a large national survey, Kråkvik et al. (2015) reported a decrease in prevalence from 14.6% (<30 years) to 2.8% (≥70 years) for auditory verbal hallucinations. Similarly, Ohayon (2000), reported a decrease in hypnagogic hallucinations, from 31.1% (15–44 years) to 15.5% (≥65 years) and for hypnopompic hallucinations, from 8.2 to 4.8%. In Soulas et al. (2016), prevalence rates of auditory hallucinations reported at interview in the age-group 60–69 years was 2.84%, 1.01% in 70–79 years, and 1.37% in ≥80. In the same study, prevalence rates of hallucinations in any modality was: 10.64% (60–69 years), 8.08% (70–79), and 8.22% (≥80). However, these differences across age groups were not statistically significant. In contrast, the prevalence of minor phenomena, such as sensed presence and passage hallucinations, increased with age in those aged 60 years and over (Soulas et al., 2016). Similarly, an age-dependent increase in the prevalence of tinnitus has been reported (Kim et al., 2015). This highlights the fact that the definition and/or type of hallucination, method of assessment and participants' personal and social characteristics, must be carefully considered when evaluating prevalence data (Larøi et al., 2005). Prevalence statistics are also complicated by variable rates of disclosure of hallucinations by older adults striving to avoid the negative stereotypes attached to these experiences, considered next.

## Stigma and distress

Studies on visual hallucinations in older adults often present qualitative evidence on the reluctance of participants to disclose their experiences for fear they are going “mad” or “demented” (self-stigma) and how they will be treated by others (public stigma) (Cox and ffytche, 2014; Pang, 2016). Similarly, studies examining post-bereavement hallucinations in older adults (a field discussed in more detail later in the article) have observed high rates of underreporting (e.g., with sometimes over 50% of people never disclosing their hallucinatory experiences to family, friends, or professionals). Of those who offered explanations why these experiences were not disclosed, participants mentioned for instance “fear to be ridiculed” (most common explanation), “too personal,” “people would not be interested,” “upset relatives if they knew,” “unlucky to talk about it” (Rees, 1971). Such beliefs may rest, in no small part, on routine portrayals of mental illness in the media (Owen, 2012). Stigma may also lead to under-reporting of hallucinatory voices in healthy adults (in all age groups), potentially preventing access to accurate information or social support and contributing to distress (Vilhauer, 2017). The addition of ageist stereotypes of cognitive incompetence (Cuddy et al., 2005; Barber, 2017) would be expected to exacerbate both self and public stigma of hallucinations in older people, but this issue has not yet been systematically explored.

Responses to hallucinatory experiences vary from person to person, and not all are found distressing. In fact, Rees (1971) reported that 68.6% of older people with post-bereavement hallucinations found the experience helpful. In general, however, compared to the literature on psychosis, fewer studies have explored the emotional reactions to hallucinations in healthy older individuals. Nonetheless, recent studies suggest that around 35% of people with Charles Bonnet Syndrome find their hallucinatory experiences distressing and are more persistent than previously thought (Cox and ffytche, 2014). Furthermore, around 10–20% of people with tinnitus report significant levels of distress, which appears to be largely independent of psychoacoustic factors and more closely related to perceived control over the experience (Wallhäusser-Franke et al., 2012). Perceived lack of control and distress typically cluster together in the experience of hallucinations in schizophrenia, suggesting shared etiological processes and the need for specific treatment (Woodward et al., 2014). A 5 year follow-up of non-psychotic adults (the majority by then aged 40–70 years) with frequent auditory verbal hallucinations, also found that distress arising from these experiences significantly predicted the need for care (Daalman et al., 2016)—a pattern that is common in younger adults with hallucinations (Johns et al., 2014). Whilst stigma may be partly responsible for the distress arising from hallucinatory experiences, a range of inter-related factors is likely to be involved. For example, Lai et al. (2016), reported that appraisals of complex visual hallucinations—as malevolent, omnipotent, or predictive of negative outcomes—were linked to high levels of distress in older, non-psychotic adults (though patients with other disorders were also included in this sample). Such findings are, however, in keeping with a larger body of evidence that negative appraisals about hallucinations are related to the level of distress experienced in both clinical and non-clinical samples (Dudley et al., 2012; Varese et al., 2016; Baumeister et al., 2017). A separate line of studies shows a consistent association between hallucinations and suicidal behaviour in younger (Connell et al., 2016; Cederlöf et al., 2017) and older samples (DeVylder et al., 2015) from the general population, probably reflecting more severe states of psychological disturbance (Honings et al., 2016).

## Factors associated with hallucinations in older adults

### Genetic factors

Multiple studies have explored the link between genetic polymorphisms (e.g., dopamine, ApoE, cholecystokinin [CCK], HOMER, tau, and COMT), and hallucinations in psychiatric disorders typical in younger (e.g., schizophrenia, bipolar disorder) and older (Alzheimer's disease, Parkinson's disease) adults. For example, changes in serotonin receptor 5HT2A expression have been implicated in the presence of visual hallucinations in Parkinson's disease, whilst the novel 5-HT2A inverse agonist, Pimavanserin, appears to be a promising treatment for these symptoms (Stahl, 2016). In general, however, conflicting results are frequent in this literature (Lenka et al., 2016). Substantially fewer studies have investigated the genetic basis of hallucinations in the general community. Nonetheless, recent evidence indicates that hallucinations are the least heritable psychotic experience in community samples (Hur et al., 2012), with no significant role for common genetic variants, at least in complex hallucinations in adolescents (Ronald, 2015; Sieradzka et al., 2015)—though a considerable influence of environmental factors. There appears to be no comparable data from older adult samples, though Lopez-Escamez et al. (2016) have argued that simple acoustic hallucinations such as tinnitus are likely to reflect a build-up of epigenetic and environmental factors over the lifetime. Consequently, although a role for rare genetic variants cannot be excluded, the literature points to a more prominent role for environmental determinants than genetic factors, in the experience of hallucinations across age all groups.

### Sensory-perceptual functioning

A common effect of ageing is the gradual decline in sensory and perceptual functioning (for a brief review see Roberts and Allen, 2016). For example, age-related hearing loss is one of the most prevalent health conditions in older adults (Mudar and Husain, 2016). UK data indicate that moderate (or worse) hearing loss affects between 39 and 45% of people aged over 60^1^, whilst visual impairment occurs in ~10% of adults aged 65–75 and 20% of those aged over 75 (Evans and Rowlands, 2004). Similar declines occur in the perception of odour, taste, and touch (Doty and Kamath, 2014; Rawal et al., 2016). In fact, dual-sensory (vision and hearing) and multi-sensory impairments are prevalent, lending support to the notion of a single, common factor underlying sensory ageing (Schneider et al., 2012; Dawes et al., 2014; Correia et al., 2016), though differential ageing of specific perceptual functions is inconsistent with this view (Billino et al., 2008).

Age-related decline in sensory functioning is often associated with a deterioration in cognitive functioning as well as poorer mental health and well-being (Hayman et al., 2007; Ciorba et al., 2012; Eramudugolla et al., 2013). Of relevance here, a recent meta-analysis of epidemiological data showed that hearing impairment (irrespective of the type, origin, or severity) is a significant risk factor for the presence of hallucinations, odds ratio = 1.4; 95 CI: 1.18–1.65 (Linszen et al., 2016). Of 5 eligible studies, 2 focused specifically on healthy older adults (70 years +) in the general community (Turvey et al., 2001; Östling and and Skoog, 2002). A major limitation in these studies is the absence of longitudinal data (Linszen et al., 2016), hence the precise nature of the relationship between auditory function and hallucinations and how they unfold in later life is unknown. In addition, details about hearing function (e.g., aetiology), co-occurring sensory impairment, and hallucinatory characteristics (such as modality) are often sparse. As a consequence, it is still unclear if hearing impairment is preferentially linked to auditory hallucinations (implicit in many explanatory models), whether hearing impairment (being the most common type of sensory impairment in old age) is associated with hallucinations in general (regardless of modality) or whether multi-sensory impairment common in older adults is associated with multi-modal hallucinations.

The idea of a modality-dependent effect for hallucinations (i.e., that auditory sensory deficits result in auditory hallucinations, visual sensory deficits result in visual hallucinations etc) is far from clear and much theoretical and empirical work, here as well, is needed. Linszen et al. (2016) in their meta-analysis (that only looked at hearing impairment and not other sensory deprivation modalities) comment that whilst this modality specific relation is implicitly suggested in contemporary models, 3 out of 5 studies included in their meta-analysis included hallucinations in other modalities as well. Further, they comment that a relation may well exist between hearing impairment and hallucinations in several different modalities, albeit most likely visual hallucinations (as they are most prevalent). They further mention that there are no current hypotheses on mechanisms underlying a connection between hearing impairment and visual hallucinations. Thus, more theoretical and empirical work is needed.

Additional evidence linking sensory functioning and hallucinations comes from clinical, neurobiological and case studies of specific types of hallucinations such as tinnitus and Charles Bonnet Syndrome (Pang, 2016; Sedley et al., 2016). Many of these reports include data from a mix of clinical and non-clinical samples (e.g., Golden and Josephs, 2015) or include selected subgroups of older adults (e.g., referred for audiometric testing) that may be vulnerable to bias (Teunisse and Olde Rikkert, 2012). In addition, though sensory impairment is common in ageing it is not inevitably associated with hallucinations, suggesting that multiple factors are involved (Roberts et al., 2010). Similar conclusions have been drawn from hallucination research on psychiatric disorders (Badcock, 2010; Waters et al., 2012; Ford et al., 2014). This multiplicity is stimulating more detailed examination of the similarities and differences in the combination of factors driving hallucinations in healthy and clinical samples (Badcock and Hugdahl, 2012) though age and clinical status are often confounded in these comparisons. For example, a recent meta-analysis of neuroimaging data on psychosis, Parkinson's disease, Charles Bonnet Syndrome and healthy hallucinators showed significant cross-study activation in auditory and visual cortices, pointing to similar mechanisms of sensory over-stimulation in the genesis of hallucinations (Zmigrod et al., 2016). The most common explanations for this increase in activation include disturbances in cortical inhibition and neurotransmitter function arising from a reduction of sensory input (Roberts et al., 2010; Pang, 2016). However, deafferentation alone may not be sufficient to account for the observed hyperexcitability associated with hallucinations: active adaptation to impairments in sensory functioning may also lead to an increased sensitivity to *intact* sensory inputs. Further understanding of how the excitatory-inhibitory balance changes with age is likely to provide valuable insights into the neural mechanisms and characteristic features of hallucinations. Sensory-perceptual impairments and hallucinations may also be related via a range of direct and indirect effects (i.e., increased demand) on cognitive and psychosocial functioning, reviewed below.

### Cognitive functioning

Cognitive functioning and, more specifically, inhibitory processes, context binding, and reality monitoring (i.e., the processes by which perceived and imagined events are discriminated; Johnson and Raye, 1981) as well as metacognition seems to play a significant role in the generation of hallucinations (Waters et al., 2012; Badcock et al., 2014; Baumeister et al., 2017). Indeed, inhibitory processes are important to suppress distractive memories, intrusive or unwanted thoughts or interfering mental images. Their dysfunction may lead to the emergence of redundant and/or irrelevant information from long-term memory into awareness, resulting in the experience of hallucinations. In support, the ability to suppress mental representation from episodic memory has been associated with the severity of hallucinations (but not delusions) in schizophrenia (Waters et al., 2003; Badcock et al., 2005; Hemsley, 2005). Similarly, Soriano et al. (2009) compared directed forgetting performance in schizophrenia patients with and without hallucination and found that patients with hallucinations presented inhibitory deficits compared to patients without hallucinations (Soriano et al., 2009). Interestingly, similar (though milder) difficulties with intentional inhibition have also been found in healthy young adults prone to hallucinations (Paulik et al., 2007, 2008), leading some authors to suggest that what may differ between clinical and non-clinical hallucinators is how they cope with and/or interpret the experience (Morrison, 2005).

Additionally, context binding deficits have been observed in schizophrenia patients and associated with more frequent hallucinations experiences, though a similar relationship has not been observed in young healthy individuals prone to hallucinations (Badcock et al., 2008). In contrast, studies have found that schizophrenia patients experiencing auditory hallucinations were more likely to incorrectly attribute the source of words they uttered themselves to the experimenter (i.e., a reality monitoring problem called an externalizing bias) compared to controls and similar effects were found in samples of non-clinical individuals prone to hallucinations (Brookwell et al., 2013) especially for negative materials compared to neutral and positive materials (Kanemoto et al., 2013). Moreover, there is some evidence that young, healthy adults predisposed to hallucinations exhibit an externalizing bias when emotionally charged material is involved and when more cognitive effort is required. Of particular relevance, compared to those with a low tendency to hallucinate, these individuals had higher scores on a scale assessing beliefs and worry about intrusive thoughts (i.e., the Meta-Cognition Questionnaire, Cartwright-Hatton and Wells, 1997) and these scores were positively associated with their source discrimination errors (Morrison et al., 2000; Larøi et al., 2004).

All these cognitive components postulated to be involved in the etiology of hallucinations are directly or indirectly affected (e.g., through reduced speed of processing, attentional resources, or working memory capacity; Park and Reuter-Lorenz, 2009) in healthy aging (Hasher and Zacks, 1988; Lustig et al., 2007; Rast, 2011). Thus, one might expect an increased likelihood of hallucinations in the elderly population compared to younger groups. That is, age-related deficits in inhibition (Lustig et al., 2007; Clarys et al., 2009; Collette et al., 2009), demanding memory processes (i.e., specific and/or distinctive recollection processes and accurate source memory, e.g., Dehon and Bredart, 2004; Dehon, 2006; Fairfield and Mammarella, 2009) as well as context binding (Mitchell et al., 2000; Piolino et al., 2010) may result in a greater likelihood of generating hallucinations in this population. However, again, only a few studies on cognitive functioning and hallucinations including healthy older adults are available and these studies are substantially limited by the fact that their samples of older adults are relatively small and/or the older adults were not their target population. That is, either some older adults were included in the general healthy sample (e.g., Stirling et al., 2007) or they served as controls for studies on hallucinations in Alzheimer's patients (El Haj et al., 2015, 2016).

Notwithstanding, in a recent set of studies, Alzheimer's disease patients and healthy older adults were asked to complete tests tapping several cognitive functions. These tasks included measures of general cognitive functioning, episodic memory, working memory (forward and backward span), verbal fluency as well as a measure of inhibition (a Stroop task; El Haj et al., 2015) or a measure of directed forgetting (El Haj et al., 2016) and a measure of auditory and visual hallucinations (i.e., 6 items from the Launay Slade Hallucinations Scale-Revised, LSHS-R; Launay and Slade, 1981). Overall, healthy older controls reported less hallucinations and had better performance on the cognitive tests than Alzheimer's disease patients. Interestingly, although most of the cognitive measures were not further included in the analyses, hallucinations were found to be mediated by inhibitory decline either as measured by the ability to supress a pre-potent response (i.e., performance in the Stroop task) or by the ability to supress memory representation (i.e., performance in a directed-forgetting task) in Alzheimer's disease (El Haj et al., 2015, 2016) but not in healthy aged-matched controls. However, the interpretation of these results needs caution due to the small samples of older adults recruited in these studies (i.e., 20–24 older control participants), the very low rates of reported hallucinations in these older adult groups that may prevent any relationship between cognitive functioning and inhibition to be observed, and the absence of younger adults that preclude any comparison based on age.

Likewise, another study on hallucination in healthy individuals that included a few older adults has shown that healthy individuals differentiated in terms of their hallucinatory experiences (i.e., high, medium, or low scores on the LSHS-R) presented distinct patterns of meta-cognitive processing—as measured on the Meta-Cognition Questionnaire (Cartwright-Hatton and Wells, 1997), but again, the data are difficult to interpret due to the inadequate sample size of older participants (Stirling et al., 2007). Other aspects that would also deserve attention are the insight associated with hallucinatory experiences in older adults and the extent to which the emotional content of hallucinations yields similar effects as those observed in young adults (Larøi et al., 2004; Kanemoto et al., 2013). Indeed, it is interesting to note that hallucinations might be associated with differing levels of insight in healthy individuals, ranging from the firm belief of the actual existence of the hallucination to the acceptance of their internal origin (see Castelnovo et al., 2015) but, again, little is known about this issue in older adults with hallucinatory experiences.

Thus, so far, the data do not seem to indicate a higher probability of hallucinations in older (compared to younger) healthy adults, but further studies are needed that focus on this population and on their cognitive particularities with large samples as there is also considerable variability in cognitive abilities with increasing age (Park and Reuter-Lorenz, 2009). In addition, the inclusion of a comparison group of younger adults would be necessary in these studies, in order to determine whether the hallucinatory experiences and their potential links with cognitive abilities that would be observed in older adults are due to direct effects of age or to other factors (e.g., reduced cognitive resources or individual differences; Park and Reuter-Lorenz, 2009). Finally, cognitive function *per se* might not be the major factor (or be sufficient alone) in hallucination generation but may instead define older adults at risk of perceptual anomalies in some situations (such as when impairment in sensory processes increases cognitive demands or when psychological and/or social factors may increase distress) or combinations of these effects. Similarly, hallucinations may be influenced by age-related effects on cognition and poor sleep quality as reviewed below.

### Sleep disturbance

Disturbance in sleep—though not exclusive to ageing—is common in older adults. Estimates suggest that around 32—36% of people over 60 years, with otherwise good health, report problems with the quantity or quality of their sleep (Foley et al., 2004; Mattis and Sehgal, 2016; Kim et al., 2017). This percentage increases in the presence of comorbid physical and mental disorders, polypharmacy, and psychosocial factors (such as social isolation, loneliness, and bereavement) and, for many, these sleep problems are chronic and undiagnosed (Miner and Kryger, 2017).

The association between sleep disturbance and psychotic disorders or symptoms has long been recognized (Kraepelin, 1919). For example, studies suggest that schizophrenia patients have more disrupted sleep-wake cycles and sleep quality than healthy controls, which exacerbates delusions and hallucinations (Monti and Monti, 2005; Afonso et al., 2011, 2014). Similarly, disturbance of sleep is common both in neurodegenerative disorders and bereavement, in which hallucinations are frequently experienced (Monk et al., 2008; Wulff et al., 2010; Burghaus et al., 2012; Llorca et al., 2016). Beyond the literature on clinical groups, surveys of the general population show that disturbances of sleep, such as insomnia, are associated with a greater likelihood of reporting current hallucinations and developing new episodes of hallucinations, suggesting that disrupted sleep may causally contribute to these experiences (Reeve et al., 2015; Sheaves et al., 2016). Whilst the relationship between sleep problems and hallucinations does not appear to be mediated by depression, anxiety or paranoia, the specific mechanistic pathways involved have not been established. However, a number of possibilities exist, including the effects of poor sleep quality on neuronal atrophy and poor cognitive functioning with increasing age (Miyata et al., 2013; Del Brutto et al., 2016). Unfortunately, large community studies have a number of limitations, including reliance on subjective report of sleep changes, lack of precision in the assessment of hallucinations or in the moderating effects of age. Consequently, a more nuanced understanding is needed of age-related changes in sleep and hallucinatory experiences. For example, it has recently been proposed that, with advancing age, hyperarousal of the sympathetic nervous system promotes the development of both tinnitus and insomnia (Wallhäusser-Franke et al., 2013). A final point worth noting is that caregivers or partners may sometimes interpret the vivid dreams reported by older adults as hallucinatory experiences. The similarities between these experiences (both involve vivid mental events) make them difficult to distinguish, though they are different, in that hallucinations entail unusual sensory experience perceived to be occurring whilst awake whilst reporting that a vivid dream felt real is based on recollection of events whilst asleep.

### Psychosocial and cultural factors

If there is strong evidence linking sensory and/or cognitive functioning and hallucinations, other factors such as loneliness and grief, which increase with age, may also affect the likelihood of hallucinations in older adults but, again, only a few empirical studies are currently available. Loneliness is the subjective experience of distress caused by lack of social support and belonging (Gierveld, 1998; Cacioppo and Cacioppo, 2014b) and is a common experience in older adults (Dykstra, 2009; Nicolaisen and Thorsen, 2014). Indeed, with increasing age, retirement, loss of partners, and/or friends, health and functional problems may limit social network size and affect social contacts which consequently increase loneliness (Aartsen and Jylhä, 2011). Increased loneliness in turn has been found to be related to depression (Prince et al., 1997), cardiovascular disease (Barth et al., 2010), and increased rates of mortality (Steptoe et al., 2013). In addition to the subjective experience of lacking social relations (i.e., loneliness; Weiss, 1973), having an objectively diminished social network (i.e., social isolation; Weiss, 1973) have been found to negatively affect cognition in old age (e.g., Bassuk et al., 1999; Fratiglioni et al., 2000; Wilson et al., 2007; Cacioppo and Cacioppo, 2014a). Recently, El Haj and colleagues specifically explored the relationship between social isolation, loneliness and hallucinations in Alzheimer's disease patients and healthy older adults. In both groups, they observed a significant correlation between hallucinations and loneliness, on the one hand, and hallucinations and social isolation on the other; although further analysis found that hallucinations were only predicted by social isolation (El Haj et al., 2016). These results are interesting as the relationship between social isolation, loneliness, and hallucinations appears to be related to ageing rather than to cognitive decline (i.e., similar correlations were observed in both healthy controls and Alzheimer's disease participants). This suggests that healthy older adults, when lacking exchange of thoughts and feelings with others or experiencing a need to communicate or express themselves, may attempt to fullfil these needs by generating internal stimulation leading to some forms of hallucination-like experiences similar to that observed in psychotic states (Hoffman, 2007, 2008). Hence, verbal and visual hallucinations may be viewed as experiences that allow individuals to escape boredom, emptiness, or distress prompted by the lack of external stimulation observed in social isolation (Hoffman, 2008). Of particular relevance for this suggestion is research showing that extreme isolation has been associated with hallucinations in prisoners and hostages confined and/or with sensory restrictions (Launay and Slade, 1981; Siegel, 1984).

Similarly, post-bereavement hallucinations have also been regarded as a compensatory mechanism for social isolation and affective deprivation or distress that may play an important part in the mourning process (Grimby, 1993). Indeed, it is not unusual for the bereaved (especially during acute grief) to sense the presence of their dead spouse, feel them watching out for or protecting them, feel they had been touched by their dead partner, to rehearse discussions, heard their voice or “speak” to them, seen them or smelled their presence (e.g., Grimby, 1993; Keen et al., 2013, see Castelnovo et al., 2015 for a recent review). Studies showed similar symptoms irrespective of gender, in sudden vs. anticipated death and religious and non-religious individuals (Wiener et al., 1996; Sacks, 2012). More relevant for the current review, these findings were largely replicated in older populations (Rees, 1971; Grimby, 1993, 1998). For instance, Rees (1971) observed that 46.7% of his participants (*n* = 293) reported post-bereavement hallucinations. Among them, adults over 40 years of age (mostly people of 60 years old and older) were more likely to be widowed and slightly more likely to see and have conversations with their dead ones than adults below 40 years of age. Similarly, Grimby (1993) observed that 82% of her older adults groups (*n* = 50) reported hallucinations and illusions following bereavement. About one half reported feeling the presence of their deceased partner (52%) and about one third reported seeing (26%), hearing (30%), and talking to her/him (30%). Additionally, marital harmony (i.e., happy marriage) was more associated with low mood, loneliness, crying, and hallucinations (for similar results see Grimby, 1998). Moreover, although manifestations of grief are influenced by social and cultural rituals, such as funerals or other customs, post-bereavement hallucinations appear to be common in many different cultures (Yamamoto et al., 1969; Lindstrõm, 1995; Grimby, 1998; Chan et al., 2005). For example, Grimby (1998) examined the incidence of hallucinations in Swedish elderly widows and widowers, within the first year after the partner's death. She found that feeling the presence of the deceased and reporting actually seeing, hearing, and talking to the deceased were the most commonly reported phenomena. Although post-bereavement hallucinations are common in many different cultures, some studies suggest that there may be important cultural differences in their prevalence, whereby certain cultures have particularly high rates of post-bereavement hallucinations. More work is needed but, for instance, Yamamoto et al. (1969) mention that prevalence rates may be higher in Japan compared to other cultures due, in part, to religions in Japan (Shintoism and Buddhism) that permit the mourner to maintain contact with the deceased, and Bell (2008) cites case reports that suggest that this phenomenon may also be particular prevalent among the Hopi Indians, Mu Ghayeb people from Oman and in Hispanic populations. Again, very little systematic work has been done in this area.

### Pharmacological issues

Polypharmacy is quite common among the elderly population as the need to treat various diseases developing with age increases. Overall, the available studies showed that nearly 50% of older adults take one or more medications that are not medically necessary (e.g., Rossi et al., 2007; Qato et al., 2008; Rambhade et al., 2012). For this reason, the clinical consequences of polypharmacy have received growing attention in recent years (Hajjar et al., 2007; Maher et al., 2014). Research has shown an increased risk of greater health costs (Hovstadius and Petersson, 2013) as well as adverse drugs events and interactions (e.g., Hohl et al., 2001) that, in turn, have huge effects on functional and cognitive capacities (Jyrkka et al., 2011) and may contribute to the development of hallucinations. Similarly, although substance abuse in older adults has received little attention in the past, illicit drugs, and substance abuse (e.g., alcohol) because of loneliness and/or boredom or overuse of prescription drugs (e.g., barbiturates) is likely to be a frequent problem in an increasing elderly population (Dowling et al., 2008). This acute or chronic abuse may be an additional factor in the development of hallucinations in this population (Wood et al., 1988; Targum, 2001). Moreover, the resulting acute withdrawal from these substances can also elicit vivid hallucinations and delusions. Whether evoked by polypharmacy or (illicit) drug abuse, close attention should be drawn on these manifestations as they may often be erroneously attributed to early signs of dementia or advancing age (Wood et al., 1988; Jyrkka et al., 2011; Rambhade et al., 2012).

## Discussion of findings

Hallucinations experienced later in life have not been a major area of interest. This first comprehensive overview the literature highlights the fact that hallucinations occur in a substantial minority of healthy older adults in the general community (McGrath et al., 2016; Soulas et al., 2016). However, public understanding of these experiences is lagging behind the evidence base, with many older (and younger) adults assuming that they are synonymous with mental illness. Consequently, more accurate information is needed in the general community to raise awareness of the varieties of hallucinatory phenomena and the many factors associated with them.

A number of issues arise from the existing literature that merit further attention. First, the considerable variation in prevalence of hallucinations in older adults is a challenge. The finding that the prevalence of auditory and visual hallucinations seems to diminish with age is surprising. Advancing age is associated with a host of risk factors for hallucinations, including impairments in sensory, cognitive, and social functioning, so the rate of hallucinations might be expected to increase. How might this discrepancy be interpreted?

One possibility is that current prevalence estimates are inaccurate. The different assessment tools used across studies (each capturing a different range of symptoms and experiences and each assessing hallucinations in different time-frames ranging from the past month to lifetime prevalence), together with the sparsity of samples aged over 80 years, makes it difficult to draw firm conclusions about the prevalence of hallucinations in different age groups. In addition, due to reluctance to disclose these experiences, it is possible that the true prevalence of hallucinations in older adults may be higher. Indeed, when reluctance to talk is overcome—as in studies of post-bereavement hallucinations—substantially higher rates have been found (Castelnovo et al., 2015).

Another possibility is that presence of individual risk factors is insufficient for hallucinations to develop. As is often noted, despite the preponderance of sensory decline with age, the majority of older adults *don't* hallucinate. Indeed, single factor models of hallucinations—in psychosis, dementia, eye-disease, and healthy hallucinators—have been superseded by more complex, multifactorial accounts (Waters et al., 2012; Collerton et al., 2016). To illustrate, occasional case reports suggest that in older people with pre-existing eye disease, the death of a spouse may increase the likelihood of Charles Bonnet Syndrome, indicating that biological and psychological mechanisms may interact (e.g., Adair and Keshavan, 1988).

**Figure 1:**
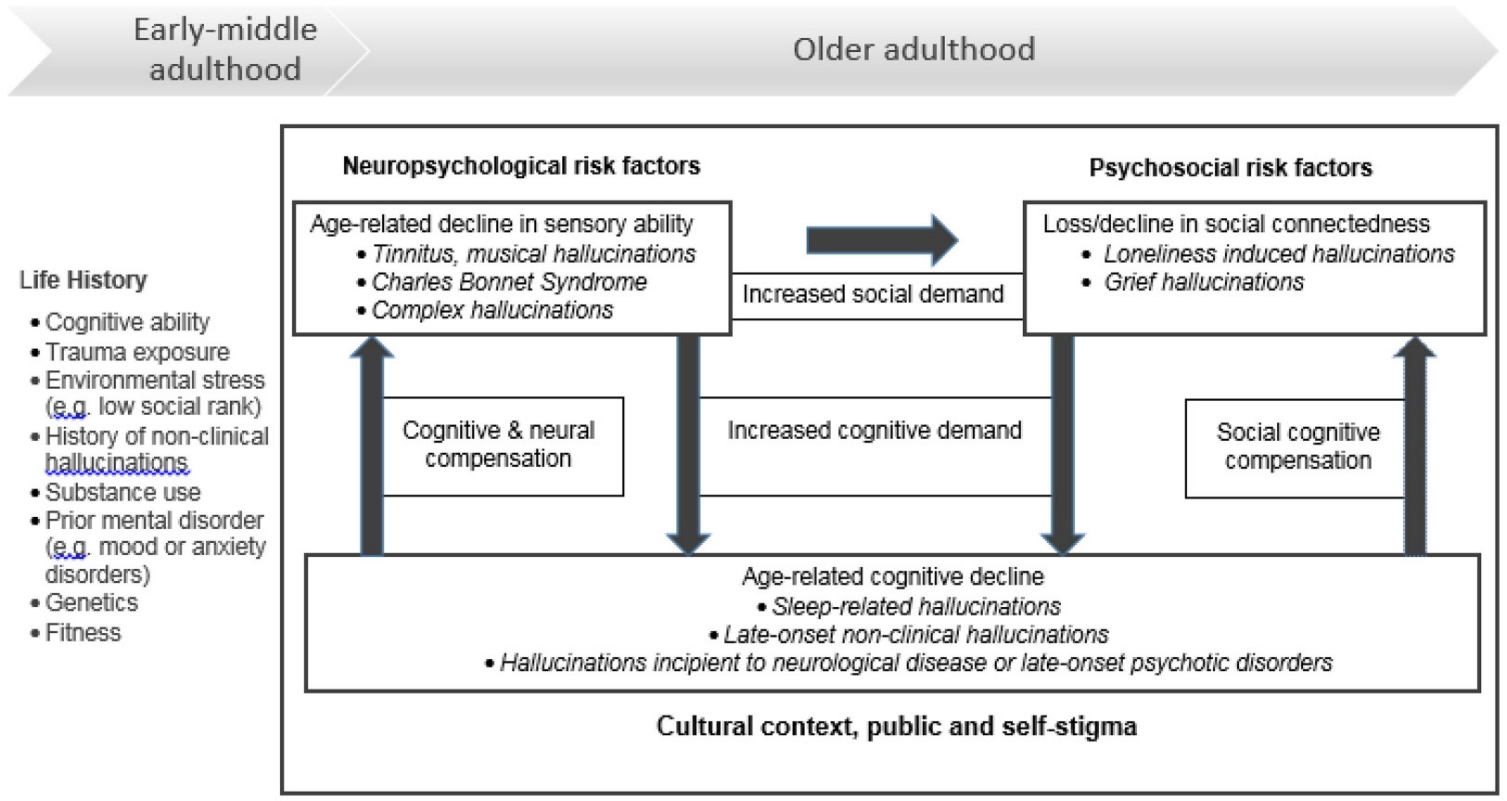
Hallucinatory experiences in healthy older adults (i.e., without psychosis, delirium, or dementia).

A third possibility governing the relationship between age and the emergence of hallucinations is that, with age, older adults actively adjust to sensory, cognitive and social decline by reorganizing neural, psychological, and social responses (Daselaar et al., 2015; Qualter et al., 2015). For example, compared to younger individuals, older adults commonly show greater task-related cortical activation (even when performance is equivalent)—argued to reflect a compensatory increase in neural response to ameliorate the adverse effects of neural decline (Reuter-Lorenz and Park, 2014). At a social level of analysis, loneliness has been shown to motivate people of all ages to re-connect with others, to fulfill the basic need for belonging (Qualter et al., 2015). Consequently, the onset of hallucinations is likely to be influenced by prior and current levels of sensory, cognitive and social (dys)function combined with the relative success of compensatory processes as well (see Figure 1).

A final consideration is whether older adults prone to hallucinatory experiences tend to die younger, reflecting an accumulation of structural and functional pathology in the central nervous system.

## Implications for aged-care services and interventions

The evidence reviewed above means that a range of aged-care health and service providers (e.g., general practitioners, pharmacists, nurses, opticians, psychologists) need to be aware that hallucinations in older adults are possible, and these may, or may not, indicate the presence of a mental disorder. Unfortunately, qualitative feedback suggests that hallucinatory experiences in the aged population are often under-recognized and/or misunderstood by many clinicians, suggesting there is significant scope for improvement in the training of both current and future professionals working with this population. In particular, service providers need to be mindful that older adults may not disclose their experience of hallucinations unless directly questioned. A wide variety of approaches have been used to assess hallucinations in older adults, though no systematic analysis of the scope and suitability of these tools for assessing these experiences in healthy older adults has yet been undertaken. In view of current evidence, screening for sleep disturbance, sensory impairments, psychosocial stressors (especially bereavement) and internalized stigma as potential causes or correlates of hallucinations, and targets for intervention, is recommended.

## Gaps in the evidence base and directions for future research

The lack of longitudinal studies is a major weakness in the current evidence base on hallucinations in older adults. Long term follow-up of healthy individuals is essential to determine the extent, and conditions under which hallucinations remit, or predict the development of future mental disease or disorders. For instance, there has been an ongoing debate as to whether visual hallucinations in Charles Bonnet Syndrome are, or are not, an early risk marker for dementia (Pliskin et al., 1996; Lapid et al., 2013). However, recent prospective data from Russell et al. (2017) point to an increased incidence of dementia in people over 65 with this syndrome. On the other hand, minor hallucinations are reported in 42% of new, untreated cases of Parkinson's disease (Pagonabarraga et al., 2016), with early evidence of visual hallucinations predicting an increased risk of dementia (ffytche et al., 2017). However, the trajectory from minor hallucinations in healthy ageing (Soulas et al., 2016) to illness onset in Parkinson's disease is unknown. Similarly, hallucinations in younger cohorts are associated with the later development of suicide attempts. However, although suicidal behaviour in older adulthood is a major problem (Conwell, 2014; Stanley et al., 2016), the long-term predictive value of hallucinations in people over 60 years has not been investigated. Notably, there is also a paucity of long term follow-up studies (see Daalman et al., 2016, as an exception) exploring hallucinations as early warning signs of late-onset psychotic disorders; consequently, the processes involved in the development of a need for care remain unclear.

A second obvious gap concerns research on post-bereavement hallucinations. Bereavement is a time when hallucinations are so common they may be viewed as the norm rather than the exception (Bell, 2008), yet studies in this area are still rare. Post-bereavement hallucinations may be particularly informative about the mechanisms driving the onset, and offset, of hallucinations, since they typically remit over time. However, although some researchers have theorized about the role of post-traumatic stress in this type of hallucination (Hayes and Leudar, 2016), empirical investigation of stress-related (biological, cognitive or social) responses is missing. In addition, understanding family members' and carer's responses to post-bereavement and other hallucinatory phenomena has received little investigation, but warrants further investigation given existing evidence that the presence of hallucinations in those with Parkinson's disease or dementia is a strong predictor of caregiver stress and nursing home admission (Oh et al., 2015; Cepoiu-Martin et al., 2016).

A final observation is the general lack of studies in this age group. Compared to the large amount of research on hallucinations in the general population, which are mostly focused on children or individuals 18–40 years, and the sizeable number of studies examining hallucinations in clinical samples (in younger and older adults)—there is a clear paucity of studies on hallucinations in healthy ageing (de Leede-Smith and Barkus, 2013; Baumeister et al., 2017). Moreover, the few studies that have examined hallucinations in community dwelling older adults have typically adopted a narrow lens (e.g., assessing sensory or cognitive factors in isolation) and relatively simple methods of investigation (e.g., self-report or behavioural measures). Future research would benefit from a more sophisticated approach examining multiple, interacting factors within the context of older people's lives. Based on the issues detailed above, some practical recommendations are proposed in Table 2 to stimulate future research.

**Table 2:** Issues for future research.

Does the prevalence of hallucinations vary in older age groups? Robust studies on prevalence are needed, stratified by age, presence/absence of sensory impairment, and inclusive of minor hallucinatory phenomena. Harmonization of assessment tools is needed to allow direct comparison of hallucinations across age groups, disorders, and time-frames. Does stigma lead to under-reporting of, or delayed help-seeking for, hallucinations in older adults and implicitly bias treatment decisions in health service providers? Are multimodal, rather than unimodal, hallucinations more common in healthy older adults and, if so, are these related to the frequent presentation of multisensory impairment in this age group? Existing explanatory accounts of hallucinations require more experimental study to test their limits and specific predictions. For example, since hearing loss is more common than visual impairment in older adults, an auditory deafferentation account might predict that auditory hallucinations will be more prevalent. In contrast, a compensatory account would argue that hearing loss leads to an increased demand on domain general executive abilities (to maintain successful communication) involved in *all* hallucinations. Hence a compensatory account might predict that hearing loss will be linked to hallucinatory experiences regardless of modality. The longitudinal trajectory of hallucinations in older adults is unknown. What is the predictive value of hallucinations for e.g., future cognitive decline, suicidal ideation, and interpersonal problems?

## Author contributions

JB and FL jointly conceived the idea for the manuscript. JB, HD, and FL all contributed to reviewing the literature, and wrote separate subsections of the manuscript. All authors provided critical commentary on all drafts, approved the final version to be published, and agree to be accountable for the content of the work.

### Conflict of interest statement

JB receives salary support from the Cooperative Research Centre-Mental Health, Carlton, Victoria, Australia. The Cooperative Research Centre-Mental Health had no role in the conduct of this review or the decision to submit the manuscript for publication. The other authors declare that the research was conducted in the absence of any commercial or financial relationships that could be construed as a potential conflict of interest.

## Footnotes

^1^Defined as >35 hearing level in decibels (dB HL) in the better ear averaged over 0.5, 1, 2, and 4 kHz frequencies (Davies, 2012).
