# Supplementary material for: Hallucinations in Healthy Older Adults: An Overview of the Literature and Perspectives for Future Research
Source: Front Psychol. 2017 Jul 7;8:1134. doi: 10.3389/fpsyg.2017.01134 (PMC5500657; doi:10.3389/fpsyg.2017.01134)
Supplement: Supplementary file 1 [file Table1.docx]

**Supplementary Table. Summary of prevalence studies reviewed.**

| **Study** | **Description** | **% prevalence** | **N** | **Mean/**  **median age (range)** | **Hallucinations measure (time frame)** | **Other comments** |
| --- | --- | --- | --- | --- | --- | --- |
| Turvey et al. (2001) | Nationwide study assessing (via telephone) hallucinations and paranoid delusions in older adults ≥ 70 years | AH and/or VH: 20% | 822 | 80 (NA) | Proxy interview, Item (“Does X ever see or hear things that are no really there – yes or no?” developed by authors | Significant association between presence of hallucinations and of delusions. Marital status, trouble with vision, cognitive impairment were associated with hallucinations and paranoid delusions |
| Livingston et al. (2001) | Randomised sample from an inner London borough of individuals ≥ 65 years | 3.9% (1 month prevalence rate of persecutory + perceptual disturbance) | 720 | 75 (65-102) | GMS (previous month) |  |
| Cole et al. (2002) | Assessed AH among older persons (≥65 yrs) attending an auditory clinic | Humming/buzzing: 5.9%; Shushing: 12.8%; Multiple sounds: 12.6%;  Beating/tapping: 10.6%; Ringing: 7.7%; Music: 2.5%; Voices: 2.5% | 125 | 77.8 (66-95) | Items developed by the authors (past month) | All participants had a certain degree of hearing impairment |
| Lyketsos et al. (2000) | Compared group with vs. without dementia | In group without dementia: 0.6% | 673 | 80.8 (NA) | NPI (past month) | The NPI measures both AH and VH in the same item |
| Larøi et al. (2005) | Compared young adult vs. older adult groups | *Older group:*  AH: 37%; VH: 33.5%;  OH: 14%; TH: 12%  *Younger group:*  AH: 19.7%; VH: 22.5%; OH: 16%; TH: 14% | 183 | 68.6 (60-75) | LSHS (“In the past”) | AH score based on replies to 3 LSHS items. VH score based on replies on 2 LSHS items. OH and TH scores based on 1 item on the LSHS per modality |
| Kråkvik et al. (2015) | General population study of AVH | *For AVH:*  <30: 14.6%  30-39: 7.8%  40-49: 6.0%  50-59: 6.4%  60-69: 4.6%  ≥70: 2.8% | *Total:* 2533  (for 6 age groups: 322 + 410 + 522 + 535 + 453 + 291) | NA (19-96) | 2 specific AVH LSHS items (“In the past”) |  |
| Ohayon et al. (2000) | Multi-site study assessing hall (in all modalities) and other experiences (HGogic, HPompic, OBE) in representative samples of the non-institutionalised general population (>15 yrs) | *HGogic*  15-44: 31.1%  45-64: 19.7%  65+: 15.5%  *HPompic*  15-44: 8.2%  45-64: 5.1%  65+: 4.8% | 13057 | NA (15-100) | Sleep-EVAL | Only age effects for HGogic and HPompic prevalence rates reported |
| Geda et al. (2008) | Compared MCI vs. normal cognitive aging | In normal cognitive aging group: 0.4% | 1590 | 79 (70-91) | NPI-Q (past month) | The NPI measures both AH and VH in the same item |
| Okura et al. (2011) | Group with cognitive impairment without dementia | 2.2% | 238 | NA | NPI (past month) | Clinically significant hallucinations correlate with significant higher mortality  The NPI measures both AH and VH in the same item |
| Soulas et al. (2016) | Assessed prevalence of hallucinations, delusions and minor phenomena in a non-demented sample ≥60 yrs | *For AH:*  60-69: 2.84%  70-79: 1.01%  ≥80: 1.37%  *For all hallucination modalities:*  60-69: 10.64%  70-79: 8.08%  ≥80: 8.22% | *Total: 313*  (for 3 age groups: 141 + 99 + 73) | NA | 10 item qualitative interview designed by authors (previous month) |  |
| Subramaniam et al. (2016) | Assessed prevalence of hallucinations and persecutory delusions in a community sample of older adults without dementia ≥60 yrs | 2.7% for *all* groups, specifically:  OH: 1.4%  AH: 1.2%  VH: 0.8%  GH: 0.05%  SH: 0.04% | *Total: 2166*  (for 3 age groups: 1449 + 535 + 182) |  | GMS (previous month) |  |
| Östling et al. (2013) | Multi-site study assessing paranoid symptoms and hallucinations in a sample of older adults without dementia ≥64 yrs | 2.6% had any psychotic symptoms (hallucinations or persecutory delusions) | Total: 8762 | NA (65-104) | GMS (previous month) in 5 sites, CPRS (past month) in 1 site | Prevalence of psychotic symptoms increased with age. Specific prevalence rates for specific symptoms and for specific age groups (64-74, 75-84, 85-94, 95-104) are not reported |
| Sigström et al. (2009) | Assessed psychotic symptoms in a population-based sample of older non-demented adults | 1-year prevalence of hallucinations:  70: 0.2%  78-82: 0.9% | NA | Participants aged 70 (women and men, N=564) and 78-82 yrs (women, N=330) were included | CPRS (past month). Key informant interviewed by telephone | “Hallucinations” consist of: commenting voices, other AH, VH, other hallucinations |
| Soares et al. (2015) | Assessed psychotic symptoms in a community-based sample of older adults ≥60 yrs | *All age groups (≥60 yrs):* AH: 7.5%;  VH/TH: 7.8%  *60-69 yrs*:  AH: 7.7%; VH/TH: 8.1%  *70-79 yrs*:  AH: 7.7%; VH/TH: 7.9%  *80-89 yrs:*  AH: 6.7%; VH/TH: 7.4%;  *≥90 yrs:*  AH: 0.0%; VH/TH: 0.0% | 1125 | NA | Hallucination items extracted from Cambridge Mental Disorders of the Elderly Examination (Do you have, or have you ever had, the experience of hearing things that other people do not?; Do you ever have the experience of seeing things other people do not?) | All participants were screened for dementia, cognitive and functional impairment |

NA: not available, NPI: Neuropsychiatric Inventory; NPI-Q: Neuropsychiatric Inventory Questionnaire; HGogic: Hypnagogic hallucinations; HPompic : Hypnopompic hallucinations; OBE: Out of body experiences; MCI: Mild cognitive impairment; LSHS: Launay-Slade Hallucinations Scale; AH: auditory hallucinations; VH: visual hallucinations; GH: gustatory hallucinations; SH: somatic hallucinations; TH: tactile hallucinations; OH: olfactory hallucinations; GMS: Geriatric Mental State schedule; CPRS: Comprehensive Psychopathological Rating Scale
